# Relationship between negative symptoms, cognitive function and social function in schizophrenia: new insight from a network analysis

**DOI:** 10.3389/fpsyt.2025.1623147

**Published:** 2025-06-26

**Authors:** Renliang Cai, Zaochen Zhu, Yan Li, Jin Fang, Chaoran Wu, Yunshan Hu, Shaotong Zhang, Chao Zhou, Xiandong Yang, Xinyu Fang, Xiangrong Zhang

**Affiliations:** ^1^ Department of Geriatric Psychiatry, The Affiliated Brain Hospital of Nanjing Medical University, Nanjing, Jiangsu, China; ^2^ Department of Psychiatry, Beijing Anding Hospital Affiliated to Capital Medical University Wuhu Hospital, Wuhu, Anhui, China; ^3^ Department of Psychiatry, The Third People’s Hospital of Qidong, Nantong, Jiangsu, China

**Keywords:** schizophrenia, negative symptoms, cognition, social function, network analysis

## Abstract

**Objective:**

This study aimed to investigate the complex relationships between negative symptoms, cognitive function, and social functioning in chronic male patients with schizophrenia, identifying core symptoms to lay a theoretical foundation for targeted interventions aimed at negative symptoms in this population.

**Methods:**

A total of 161 male schizophrenia patients were included, categorized into deficit syndrome (DS) and non-DS groups using the Chinese version of the Schedule for the Deficit Syndrome (SDS). Social functioning was assessed with the Scale of Social Function in Psychosis Inpatients (SSPI), while a battery of neurocognitive tests measured cognitive domains, including sustained attention, cognitive flexibility, ideation fluency, and visuospatial memory. Network analysis was employed to construct an integrated network of negative symptoms, cognitive function, and social functioning, aiming to identify the most central and bridge symptoms within these networks.

**Results:**

Our study indicated that DS patients performed worser in cognitive function and social functioning than non-DS patients. The network analysis demonstrated that “intensity of pleasure during activities (B1)” in the negative symptoms was the most central node. The most prominent bridge node was SSPI, with impact indices of 0.55.

**Conclusion:**

Our findings provided evidence revealing a closer connection between negative symptoms, cognitive function, and social functioning. In light of these findings, precise targets for pharmacological treatment, psychotherapy and physical therapy are identified for patients with schizophrenia.

## Introduction

Schizophrenia is considered as one of the most severe psychiatric disorders. A significant number of those who develop this condition do not fully recover, and even for those with positive outcomes, the diagnosis can lead to profound changes in life, including but not limited to social isolation and stigma (1). Schizophrenia has been always recognized as a core set of clinical features that characterize the disorder. These features encompass positive symptoms, negative symptoms, and cognitive impairments (2). In the advanced stages, negative symptoms and cognitive can be more severe and lead to great economic burdens on families and society (3). Consequently, there has been a growing focus on targeting the negative symptoms and cognitive impairments of schizophrenia as primary objectives in psychological treatment.

Negative symptoms consist of lack of volition, reduced speech output, and flattening of affect, which occur in up to 60% of schizophrenia patients (4). It carried a high burden of disease in schizophrenia, such as liability for illness onset (5), decreased psychological well-being (6), poor social functioning (7), impaired role functioning (8), and reduced rates of recovery (9). Despite their substantial impact, effective interventions for negative symptoms are lacking, and these symptoms typically do not respond to antipsychotic therapy (10). The limited advancement in developing effective treatments can be attributed, in part, to an incomplete understanding of the latent structure of negative symptoms and the mechanisms underlying their various components.

Deficit syndrome (DS) is regarded as a pathophysiological subtype of schizophrenia, characterized by primary and persistent negative symptoms (11). The presence of DS is believed to influence functional outcomes and the course of the illness, with stronger associations to persistent impairments in social, occupational, and symptom-related outcomes compared to non-deficit syndrome (9). This has spurred growing interest in exploring the underlying structure of negative symptoms. Recent research reported significantly elevated CRP levels in DS patients compared to non-DS patients (12). Currently, two-factor and five-factor models are widely acknowledged in the study of negative symptoms. The five-factor model, reflected in the Brief Negative Symptom Scale (BNSS) (13) and Clinical Assessment Interview for Negative Symptoms (CAINS) (14), encompasses affective flattening, alogia, avolition, asociality, and anhedonia. In contrast, several studies employing exploratory factor analysis on negative symptom scales have consistently identified two factors: motivation and pleasure deficits (MAP) and expressivity deficits (EXP) (15). The two-factor model is supported by researchers for its potential to uncover the nature of negative symptoms and identify therapeutic targets (16). Neuroimaging studies further back the two-factor model, demonstrating that MAP and EXP are differentially associated with functional outcomes and involve distinct mechanisms and pathways (17). However, a recent study analyzing the factor structures of the BNSS, CAINS, and Scale for Assessment of Negative Symptoms (SANS) suggested that the two-factor model fails to fully capture the complexity of negative symptoms in schizophrenia (18). Instead, the authors concluded that the original five-factor model more accurately represents the latent structure of negative symptoms. Some subsequent studies from different cultures have confirmed the existence of the hierarchical model through the application of confirmatory factor analysis (19, 20) and network analysis (21). Clarifying the latent structure of negative symptoms is important, because it provides researchers with valuable insights into relevant pathophysiological mechanisms. Some preliminary studies have indicated that individual negative symptom domains have different pathophysiological correlations (22), suggesting that further research is needed.

Cognitive dysfunction is also a core feature of psychosis that is strongly correlated with poorer social outcomes, particularly a decrease in patients’ quality of life. These deficits often emerge nearly a decade before the first onset of psychosis and tend to worsen as the disease progresses (23). A recent network analysis further confirmed that negative symptoms and cognitive dysfunction are the most significant and influential factors affecting social functioning (24). Another meta-analytic study also suggested that cognitive dysfunction is a key predictor of both current and future psychosocial functioning (25). Because of the complex relationship between negative symptoms, cognitive functioning, and social functioning, understanding their internal structure is crucial for identifying therapeutic targets for negative symptoms. Many previous studies examining these relationships simply employed correlation and regression analyses, without considering the internal relationships between the different factors of negative symptoms and the various cognitive dimensions (26).

To address these issues, we introduced network analysis, a mathematical approach designed to evaluate complex systems. Network analysis focuses on the interrelationships among system components, making it particularly well-suited for estimating the latent structure of constructs. Instead of analyzing each factor individually as a single effect of a causal disorder, network analysis considers the interactions between all factors. This approach ensures that no factor is examined in isolation, but rather in the context of its influence on and by all other factors (27). Network analysis is a data-driven approach that does not rely on predefined hypotheses about causal relationships between variables. In contrast to traditional methods, such as structural equation modeling, which require assumptions about causality, network analysis bypasses these constraints. Moreover, network analysis allows for the estimation and visualization of the overall patterns of interrelationships among variables as network structures, while also generating useful indices to assess the centrality of each variable (28). Recent network analyses have examined BNSS structure and cognition-functioning links, supporting a data-driven approach. Our study builds on this within the evolving landscape of data-driven models of psychopathology (29, 30). As a result, network analysis holds significant potential for uncovering the complex interactions between negative symptoms, cognitive function, and social functioning in patients with schizophrenia.

In summary, we utilized network analysis to investigate the intricate relationships among negative symptoms, cognitive function, and social functioning in chronic male patients with schizophrenia. Only male participants were included to control for sex-based differences in schizophrenia symptomatology and cognitive profiles, which may confound network estimation (31). Additionally, we aimed to identify the most central nodes within the resulting network using centrality indices.

## Methods

### Participants

A total of 161 male patients diagnosed with schizophrenia were recruited from the inpatient department of the psychiatric rehabilitation unit at Yangzhou Wutaishan Hospital in Jiangsu Province. Diagnosis of schizophrenia was confirmed according to the criteria outlined in the Diagnostic and Statistical Manual of Mental Disorders, Fourth Edition (DSM-IV). Eligibility for participation required patients to be between the ages of 20 and 65, have been on a stable regimen of oral antipsychotic medication for at least 12 months, and demonstrate stable schizophrenia symptoms. Exclusion criteria included the presence of physical comorbidities, substance abuse or dependence, or any prior receipt of physical treatments such as electroconvulsive therapy. No missing data were present in the final sample, as incomplete cases were excluded during screening. The study was approved by the Institutional Ethical Committee for Clinical Research, and all participants provided written informed consent.

### Subtypes of DS and NDS patients

The Chinese version of the Schedule for the Deficit Syndrome (SDS) was used to classify patients into deficit syndrome (DS) and non-deficit syndrome (NDS) (32), and is currently regarded as the ‘gold standard’ for assessing deficit symptoms. Interviews should be conducted when the interviewee’s mental state is clinically stable. The SDS evaluates six specific symptoms: restricted affect, poverty of speech, diminished sense of purpose, reduced social drive, curtailed interest, and limited emotional range. A diagnosis of deficit syndrome is confirmed if two or more of these symptoms are present in the SDS assessment. Furthermore, the symptoms must have persisted for at least one year before the evaluation (33, 34). The DS/NDS classification was employed solely for sample characterization. Due to the small sample size, we did not perform network analysis for DS and NDS separately. All participants were included in the network analysis.

### Clinical assessment

#### The Brief Negative Symptoms Scale

The BNSS is a 13-item interview-based rating scale organized into six subscales (13). It evaluates five symptoms that are widely recognized as negative symptoms of schizophrenia: anhedonia, asociality, avolition, blunted affect, and alogia. A sixth subscale, which assesses psychic distress (worrying), was also included. These symptoms are grouped into two factors. The MAP factor consists of three subscales that assess deficits in social functioning, including anhedonia, avolition, and asociality. The EXP factor includes two subscales that assess emotional expression, namely blunted affect and alogia. The BNSS was administered by trained raters (psychiatrists with >5 years’ experience), with inter-rater reliability assessed via intraclass correlation coefficient (ICC > 0.75). All items are rated on a 7-point scale (0–6), with anchor points ranging from the absence of symptoms (0) to severe symptoms (6).

A series of classical neurocognitive tests, encompassing a wide range of cognitive functions, was used to assess the neurocognitive abilities of all participants, including the Digit Vigilance test (DVT), Animal Naming Test (ANT), Controlled Oral Word Association Test (COWAT), a Block Design (Wechsler adult intelligence scale- Chinese Revision, WAIS-RC), Trail Making Test-A, B (TMT-A,B), Stroop Color-Word test (SCWT) and Spatial Processing (35–37). Based on earlier studies, these tests were categorized into the cognitive domains of sustained vigilance/attention (Stroop words only, colors only, TMT-A, and DVT), cognitive flexibility (Stroop color/word interference test and TMT-B), ideation fluency (COWAT and ANT), and visuospatial memory (spatial processing test and WAIS-RC) (11).

#### Scale of Social Function in Psychosis Inpatients

The SSPI was used to assess the social functioning of patients (38, 39). It consists of 12 items based on the actual living conditions and rehabilitation content for inpatients with psychosis. These items were grouped into three factors based on similar qualities. The factors evaluate life skills, mobility, social competence, and social activity skills, with each factor containing four items. Each item is rated on a 5-point scale (0 = extreme impairment; 1 = severe impairment; 2 = moderate impairment; 3 = mild impairment; 4 = no impairment). The total score ranges from 0 to 48, with higher scores indicating better social functioning. The SSPI total score was used to construct network structure, individual items were not included to avoid model complexity.

### Demographic data analysis

The data analyses were conducted using SPSS V.23.0 software. The independent t-test was used to compare age, education level, BMI, total disease course, negative symptoms, cognitive function and SSPI between the DS and non-DS patients.

### Network analysis

We used simple descriptive statistics (proportion, mean, and standard deviation) to present the demographic and clinical characteristics of the sample. The network structure was estimated for all the items (13 items of the BNSS, 9 items of neurocognitive tests and 3 items of the SSPI). A network represents a system of nodes that are connected in various ways (40, 41). In network analysis, edges represent the connections between nodes. Regularized partial correlations were estimated using the graphical least absolute shrinkage and selection operator (GLASSO) algorithm to minimize false-positive connections. For our study, the nodes consisted of various items from the BNSS, neurocognitive tests, and the SSPI, while the edges corresponded to the partial correlation coefficients between these items. The R package *qgraph* was used to construct a weighted undirected network, where the strength of the correlation between two items was represented by the thickness of the connecting line. To evaluate the significance of each item, we employed three centrality measures: node strength (the sum of all weighted connections), closeness (the multiplicative inverse of the total length of the shortest paths between the node and all other nodes), and betweenness (the number of times a node appears on the shortest path between two other nodes). Bridge strength quantifies a node’s capacity to connect distinct clinical domains (e.g., negative symptoms ↔ cognition), calculated as the sum of edge weights linking different communities. We removed the nodes connections within the same clinical domains and reconstructed the network structure of the bridge centrality to measure the bridge symptom. Additionally, to assess the stability of these centrality indices, we calculated the correlation stability (CS) coefficient. Reliable centrality estimates require a CS coefficient greater than or equal to 0.25, with values above 0.5 considered ideal.

Afterwards, we evaluated node centrality based on node strength to understand the structural significance of each node in the network (40, 41). Node strength was selected because it indicates the direct impact of a node on the entire network. To assess the network’s stability, we used the bootnet R package, which generated random subsamples with progressively smaller sizes from the entire population.

## Result

### Characteristic of the participants

A total of 161 schizophrenia patients were included in this study, with 70 patients in the DS subgroup and 91 in the non-DS subgroup. The overall characteristics of the patients are presented in **Table 1**. No significant differences were observed between the two subgroups in terms of age, education level, total disease course, or body mass index (BMI) (all P > 0.05). However, DS patients exhibited more severe negative symptoms across all 13 items of the BNSS, as well as greater impairment in all four domains of cognitive function and social functioning compared to non-DS patients (all P < 0.001).

**Table 1:** Comparisons of demographic and clinical characteristics between DS schizophrenia patients and non-DS schizophrenia patients.

Characteristic	DS patients (N=70)	NDS patients (N=91)	F/t	P
Age	50.26 ± 8.37	49.88± 7.80	0.295	0.768
Education level (year)	8.30 ± 2.80	9.05 ± 2.57	1.799	0.077
BMI	24.05 ± 3.57	24.86 ± 3.13	1.540	0.126
Total disease course (year)	27.93 ± 7.69	26.54 ± 8.03	1.109	0.269
B1: Intensity of pleasure during activities	3.64 ± 0.12	1.71 ± 0.10	12.145	<0.001
B2: Frequency of pleasure during activities	3.56 ± 0.14	1.67 ± 0.10	10.807	<0.001
B3: Intensity of expected pleasure from future activities	3.66 ± 0.16	1.50 ± 0.11	11.352	<0.001
B4: Distress	4.16 ± 0.16	2.28 ± 0.11	9.584	<0.001
B5: Asociality: behavior	3.50 ± 0.13	1.56 ± 0.13	10.669	<0.001
B6: Asociality: internal experience	3.94 ± 0.14	1.66 ± 0.11	12.865	<0.001
B7: Avolition: behavior	3.54 ± 0.14	1.52 ± 0.11	11.536	<0.001
B8: Avolition: internal experience	3.90 ± 0.13	1.80 ± 0.11	12.286	<0.001
B9: Facial expression	3.02 ± 0.15	1.21 ± 0.10	10.200	<0.001
B10: Vocal expression	2.61 ± 0.16	0.95 ± 0.10	8.850	<0.001
B11: Expressive gestures	2.96 ± 0.16	1.07 ± 0.10	9.972	<0.001
B12: Quantity of speech	2.96 ± 0.16	1.24 ± 0.10	9.273	<0.001
B13: Spontaneous elaboration	3.47 ± 0.18	1.56 ± 0.12	8.886	<0.001
BNSS total score	44.93 ± 13.03	19.70 ± 9.85	13.495	<0.001
Y1: Sustained attention	12.97 ± 4.08	5.36 ± 3.39	12.599	<0.001
Y2: Cognitive flexibility	5.99 ± 2.37	2.45 ± 1.64	10.672	<0.001
Y3:Ideation fluency	3.94 ± 1.19	1.66 ± 1.06	12.865	<0.001
Y4: Visuospatial memory	7.01 ± 2.43	3.08 ± 1.86	11.265	<0.001
SSPI total score	18.74 ± 8.72	31.81 ± 6.73	10.375	<0.001

### Estimation of the network

**Figure 1:**
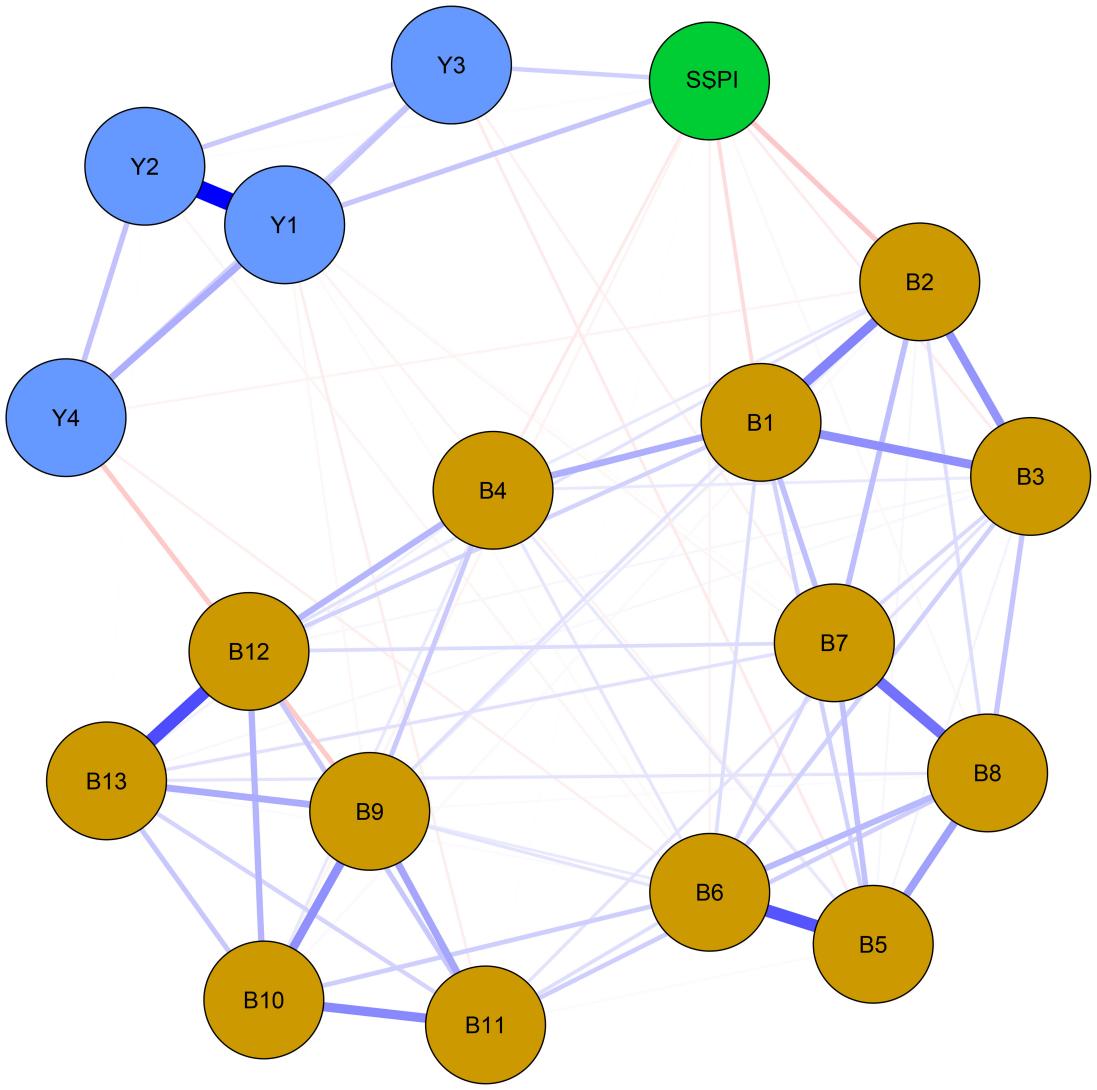
Network structure of negative symptoms, cognitive function and social function in schizophrenia. Blue edges denote positive partial correlations, red edges denote negative correlations. Thickness corresponds to correlation strength. Nodes are color-coded by domain: Yellow = Negative symptoms (B1-B13: 13 items of BNSS); Blue = Cognitive function (Y1: sustained vigilance/attention; Y2: cognitive flexibility; Y3: ideation fluency; Y4: visuospatial memory); Green = Social function (SSPI: total score of SSPI).

The regularized partial correlation network about negative symptom, cognitive function and social functioning in male schizophrenia patients is shown in **Figure 1**. The network consisted of 153 regularized partial correlations, with 88 edges showing positive associations. Blue edges indicate positive correlations, while red edges represent negative correlations. The thickness of the edge reflects the magnitude of the correlation.

Four groups of symptoms in the items of the functional subscale show obvious regularized partial correlation. They are “sustained vigilance/attention (Y1)” and “cognitive flexibility (Y2)”, “quantity of speech (B12)” and “spontaneous elaboration (B13)”, “asociality behavior (B5)” and “asociality inner experience (B6)”, and “avolition behavior (B7)” and “avolition inner experience (B8)”, with corresponding regularized partial correlation coefficients of 0.533, 0.371, 0.355 and 0.295, respectively. Similarly, “intensity of pleasure during activities (B1)”, “frequency of pleasure during activities (B2)” and “intensity of expected pleasure from future activities (B3)” as well as “facial expression (B9)”, “vocal expression (B10)” and “expressive gestures (B11)” also show some regularized partial correlation. The regularized partial correlation coefficients between the two of them are around 0.25.

### Centrality analysis results

**Figure 2:**
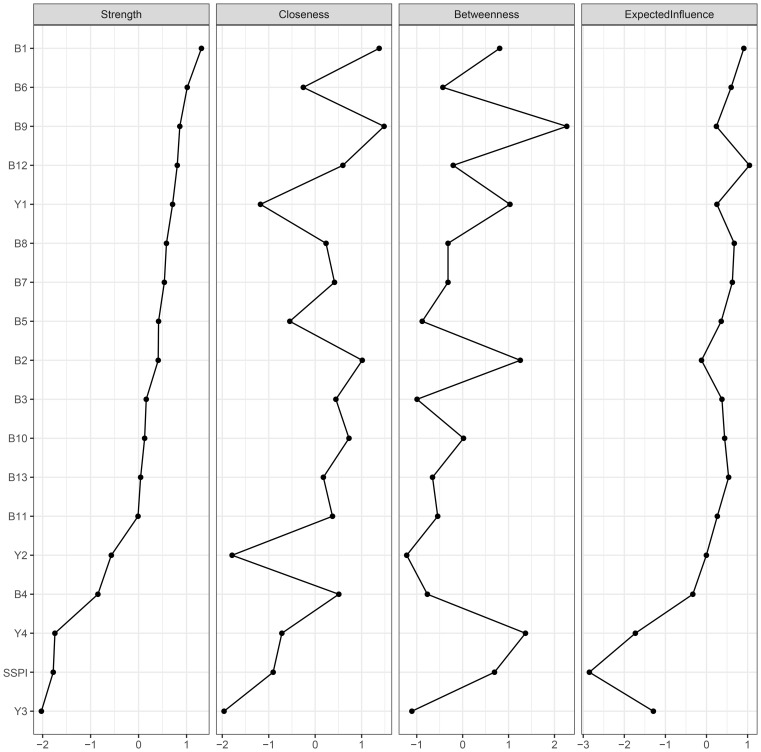
Centrality plot of the regularized partial correlation network. B1-B13: 13 items of BNSS; Y1: sustained vigilance/attention; Y2: cognitive flexibility; Y3: ideation fluency; Y4: visuospatial memory; SSPI: total score of SSPI.

For network centrality, “intensity of pleasure during activities (B1)” in the negative symptoms was the most central node, followed by “asociality inner experience (B6)”. Therefore, these two nodes may be central to the association among negative symptoms, cognitive function, and social functioning in chronic male patients with schizophrenia. The node “ideation fluency (Y3)” in the cognitive function had the lowest strength and expected influence (**Figure 2**).

**Figure 3:**
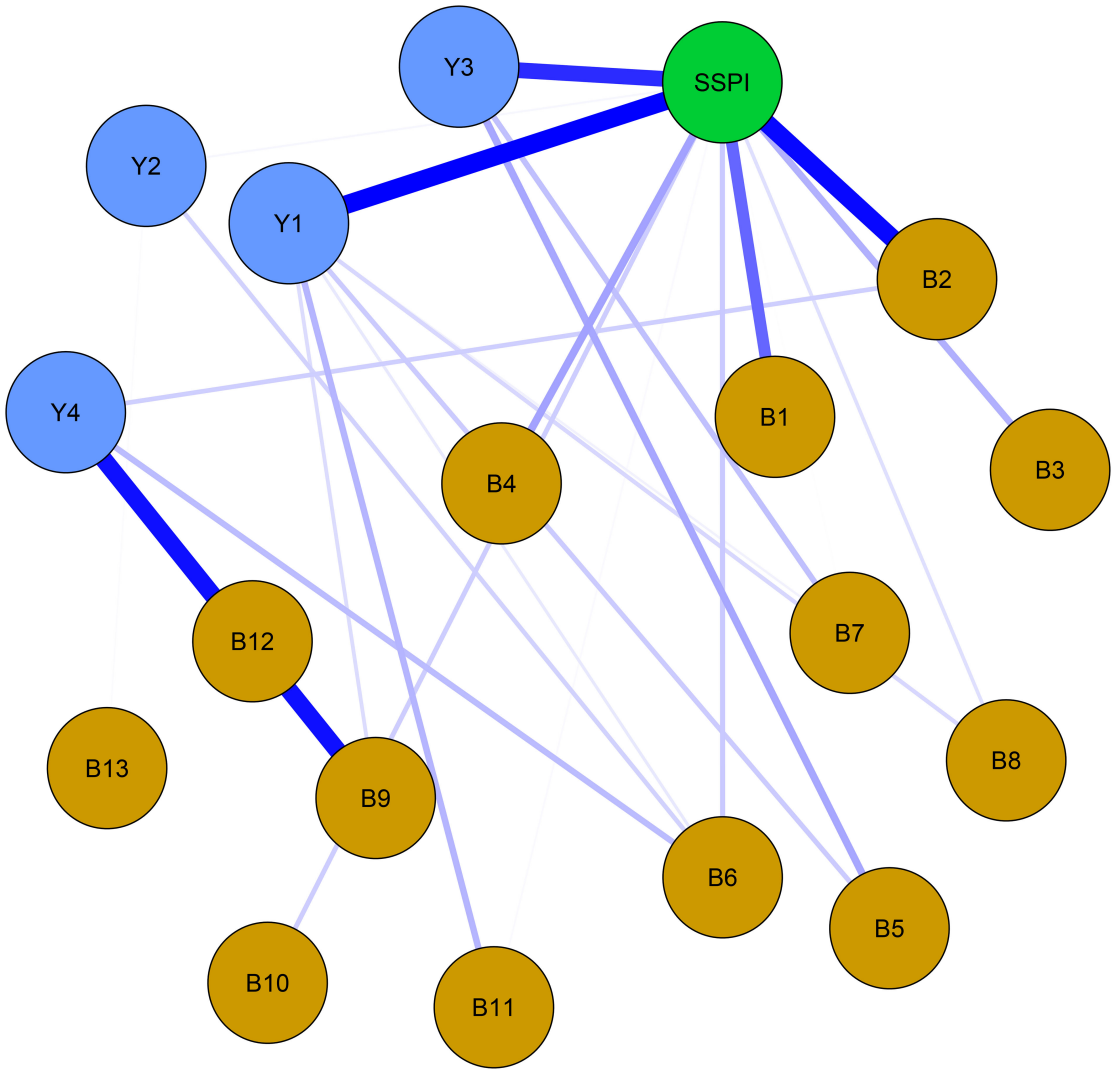
Network structure of bridge centrality with negative symptoms, cognitive function and social function in schizophrenia. Thickness corresponds to correlation strength. Nodes are color-coded by domain: Yellow = Negative symptoms (B1-B13: 13 items of BNSS); Blue = Cognitive function (Y1: sustained vigilance/attention; Y2: cognitive flexibility; Y3: ideation fluency; Y4: visuospatial memory); Green = Social function (SSPI: total score of SSPI).

**Figure 4:**
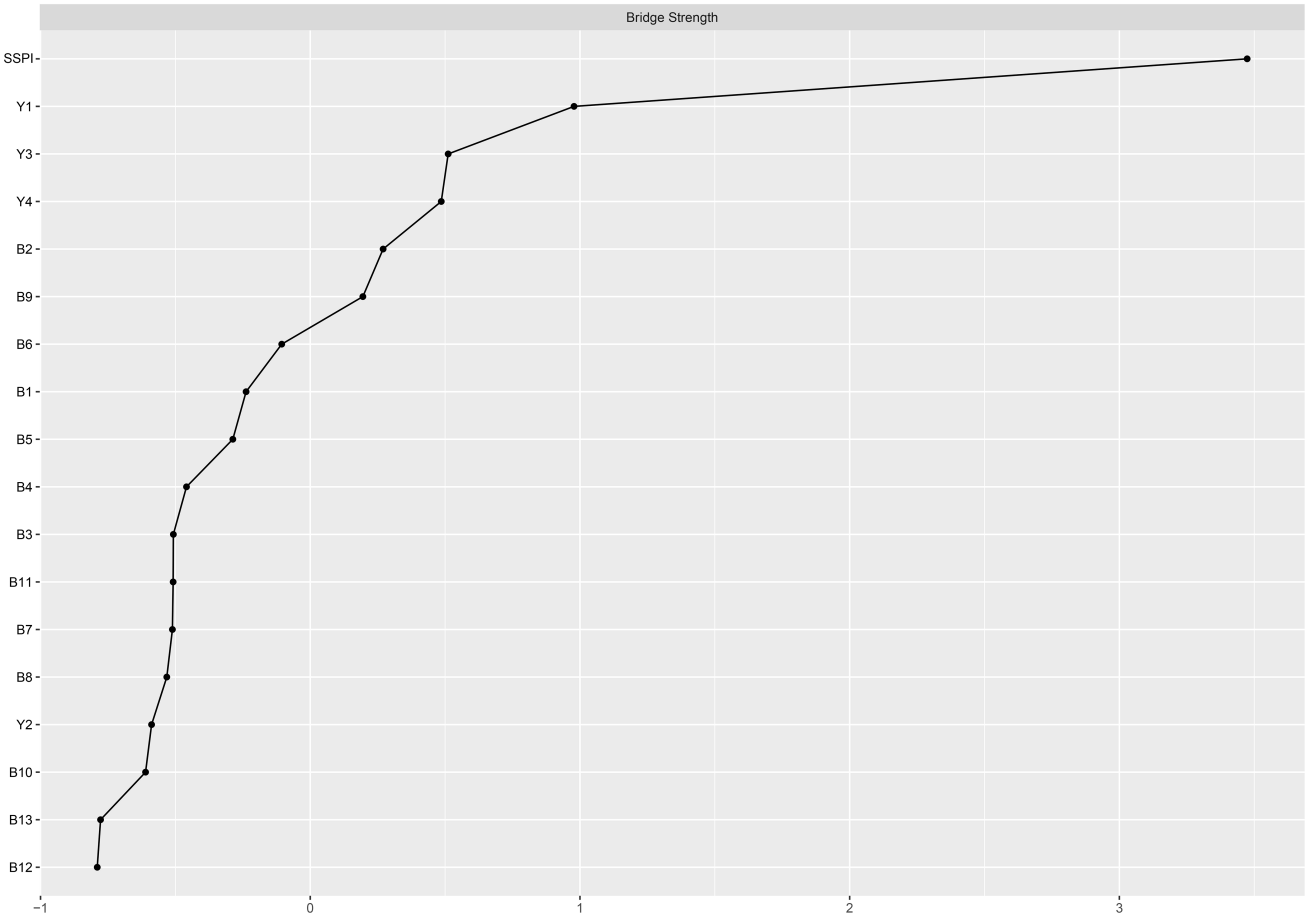
Bridge centrality plot of negative symptoms, cognitive function and social function in schizophrenia. B1-B13: 13 items of BNSS; Y1: sustained vigilance/attention; Y2: cognitive flexibility; Y3: ideation fluency; Y4: visuospatial memory; SSPI: total score of SSPI.

The larger value of the bridge strength, the more it represents a bridge symptom in the network. The most prominent bridge node was SSPI, which had significantly strong associations with negative symptoms and cognitive function. (**Figure 3**) “Frequency of pleasure during activities (B2)” showed the highest bridge strength among the negative symptoms, and “sustained vigilance/attention (Y1)” was the strongest bridge in cognitive function connected with the other clusters (**Figure 4**).

Among them, the bridge strength of SSPI is 0.55, the bridge strength of “frequency of pleasure during activities (B2)” is 0.138, the bridge strength of “sustained vigilance/attention (Y1)” is 0.388. Nodes with high Bridge strength displaying high topological centrality in the symptom network. They are key nodes in the network structure associated with male chronic schizophrenia symptomatology.

### Network stability and accuracy

**Supplementary Figure S1** displays the accuracy of the bootstrap method in obtaining edge weights. The narrow confidence interval range indicates that the edge weights have sufficient accuracy.

The network accuracy and the stability of centrality indices are presented in **Supplementary Figure S2**. The correlation stability (CS) coefficient for strength is 0.596, for edge is 0.671 and for bridge strength is 0.516, suggesting both are sufficient stability. However, the CS coefficients for closeness and betweenness are 0.05 and 0.05 respectively, falling below the recommended minimum threshold (ie, p > 0.25).

## Discussion

In the current study, we found that DS patients exhibited poorer performance across several domains. Previous neuroimaging studies have reported reduced gray matter volume in the right temporal lobe, decreased cortical thickness in the left temporoparietal junction (42), and abnormal nodal connectivity in the right inferior temporal gyrus (43) in DS patients. These structural abnormalities in the temporal lobes may be related to an early-onset, nonprogressive developmental process, which interferes with the acquisition of basic cognitive and social skills from childhood, potentially explaining the poor premorbid adjustment and social cognitive deficits observed in this subgroup (44). These neuroimaging findings align with the results of our study. Therefore, DS may represent a distinct subtype of schizophrenia, and further evidence is needed to support this hypothesis.

For the network analysis, negative symptoms, cognitive function, and social functioning were used as nodes to construct the network for chronic male patients with schizophrenia. The results indicate that the strongest connection is observed between “sustained vigilance/attention (Y1)” and “cognitive flexibility (Y2)”, “quantity of speech (B12)” and “spontaneous elaboration (B13)”, “asociality behavior (B5)” and “asociality inner experience (B6)”, and “avolition behavior (B7)” and “avolition inner experience (B8)”. “Intensity of pleasure during activities (B1)” and “asociality inner experience (B6)” are the central symptoms of the network. The bridge node in network is SSPI.

In our regularized partial correlation network, the stronger connections are between Y1-Y2, B12-B13, B5-B6 and B7-B8. Moreover, B1-B2-B3 and B9-B10-B11 also show some regularized partial correlation. This suggests that our results support five factors structure of negative symptoms. It can also be observed that the clusters of negative symptoms are divided into two groups, consistent with the previous categorization of the MAP and EXP factors. However, the connection between MAP and EXP is weak, and further investigation is needed to determine whether the internal structure of negative symptoms align with the two second-order factors (MAP and EXP) and five first-order factors (alogia, blunted affect, anhedonia, avolition, asociality).

In the centrality analysis results, “intensity of pleasure during activities (B1)” is the central node of the network. This node is an important part of anhedonia, which is traditionally defined as an inability to experience pleasure (45). Anhedonia is a deficit in the ability to experience positive reinforcement, including the pleasure typically derived from social interactions, and is manifested as a low social drive. It is often regarded as one of the core symptoms of schizophrenia, which aligns with our findings. Furthermore, converging evidence suggests that anhedonia is a persistent phenotype, associated with schizotypal traits in non-clinical populations, and serves as a strong predictor of schizophrenia-spectrum disorders and their related social impairments (46, 47).

From the bridge analysis results, “SSPI” is the most important bridge node of our network. This node exhibits the most extensive and strongest connections with other nodes in the network. As a bridge node, it is statistically associated with co-occurring symptom activation, propagating the effect throughout the entire system (48). From the perspective of the entire network, the nodes that are most closely to the “SSPI” are “intensity of pleasure during activities (B1)”, “frequency of pleasure during activities (B2)”, “sustained vigilance/attention (Y1)” and “ideation fluency (Y3)”, suggests trans-domain mechanisms. This may inform interventions targeting anhedonia to improve social functioning, such as cognitive-behavioral therapy or pharmacological agents. The activation of “SSPI” will have a positive effect in regulating negative symptoms, especially in anhedonia. However, it can also worsen cognitive functioning, particularly in “sustained vigilance/attention (Y1)” and “ideation fluency (Y3)” in individuals with schizophrenia. Social impairment is common in schizophrenia and contributes significantly to its morbidity (49). Furthermore, it shows minimal response to treatment, and always predates the onset of psychosis (50, 51). Social impairment is a characteristic of clinical high-risk individuals, occurring at levels comparable to those seen in established schizophrenia. Several previous studies have indicated that social impairment across the schizophrenia spectrum is strongly associated with negative symptoms, along with cognitive deficits (52). Our results go further than previous studies in demonstrating that social disorders are more related to anhedonia in negative symptoms and to sustained vigilance/attention and ideation fluency in cognitive function.

This study has several limitations. First, it is cross-sectional in design and lacks a longitudinal component. Future research should incorporate regular follow-up and data collection at multiple time points to allow for a more comprehensive temporal analysis. Second, the study included only male schizophrenia inpatients, which may limit the generalizability of the findings to female patients or outpatients. Third, the neurocognitive tests used did not encompass all aspects of cognitive function, underscoring the need for more comprehensive tools in future studies to fully assess cognitive abilities. Fourth, although this study focuses on negative symptoms, cognitive function, and social functioning in schizophrenia, our understanding of the underlying mechanisms remains limited, even with the use of network analysis. Fifthly, while network analysis identifies central nodes that may inform interventions, it operates at the phenomenological level and does not directly probe underlying biology. Future studies should integrate multimodal data (e.g., EEG, genetics) to validate these nodes as true pathophysiological targets. Sixthly, our study used SSPI for social functioning; future work should incorporate real-world outcomes (e.g., employment status) to validate findings and enhance ecological validity. Crucially, symptom networks derived from cross-sectional data reveal statistical associations, not causal pathways. While highly connected nodes may represent clinically relevant intervention targets, these nodes reflect downstream manifestations of the disorder, not its underlying etiology. Their role in symptom perpetuation requires validation through longitudinal designs tracking symptom dynamics. Finally, the sample size was relatively small. In summary, future longitudinal studies with larger and more diverse samples, including both male and female patients, as well as more comprehensive assessment tools, are needed to further explore the associations among negative symptoms, cognitive function, and social functioning in schizophrenia.

This study investigated the organization of negative symptoms, cognitive function, and social functioning within a network framework, establishing a preliminary relationship network among these domains in schizophrenia. Social functioning, widely interconnected with other nodes, can activate and propagate effects across the entire network (53, 54). Targeting this node through intervention may effectively limit the spread of negative symptoms and help prevent cognitive decline. Our findings offer a theoretical basis for developing interventions for negative symptoms in schizophrenia.

## Data Availability

The original contributions presented in the study are included in the article/**Supplementary Material**. Further inquiries can be directed to the corresponding authors.

## Supplementary material

Supplementary Figure 1 Results of the accuracy of edge weights.

Supplementary Figure 2 The stability of network structure by case-dropping bootstrap. **(A)** stability of strength centrality; **(B)** stability of edge centrality; **(C)** stability of bridge strength.

## References

[1] Marwaha S Johnson S Bebbington P Stafford M Angermeyer MC Brugha T. Rates and correlates of employment in people with schizophrenia in the UK, France and Germany. Br J Psychiatry. (2007) 191:30–7. doi: 10.1192/bjp.bp.105.020982 17602122

[2] Jauhar S Johnstone M Mckenna PJ. Schizophrenia. Lancet. (2022) 10323:399. doi: 10.1016/S0140-6736(21)01730-X 35093231

[3] Rund BR Barder HE Evensen J Haahr U t. V. Hegelstad W Joa I. Neurocognition and duration of psychosis: A 10-year follow-up of first-episode patients. Schizophr Bull. (2015) 42:87–95. doi: 10.1093/schbul/sbv083 26101305 PMC4681546

[4] Correll CU Schooler NR. Negative symptoms in schizophrenia: A review and clinical guide for recognition, assessment, and treatment. Neuropsychiatr Dis Treat. (2020) 16:519–34. doi: 10.2147/NDT.S225643 PMC7041437 32110026

[5] Piskulic D Addington J Cadenhead KS Cannon TD Cornblatt BA Heinssen R. Negative symptoms in individuals at clinical high risk of psychosis. Psychiatry Res. (2012) 196:220–4. doi: 10.1016/j.psychres.2012.02.018 PMC4119605 22445704

[6] Strauss GP Sandt AR Catalano LT Allen DN. Negative symptoms and depression predict lower psychological well-being in individuals with schizophrenia. Compr Psychiatry. (2012) 53:1137–44. doi: 10.1016/j.comppsych.2012.05.009 22770716

[7] Kalin M Kaplan S Gould F Pinkham AE Penn DL Harvey PD. Social cognition, social competence, negative symptoms and social outcomes: Inter-relationships in people with schizophrenia. J Psychiatr Res. (2015) 68:254–60. doi: 10.1016/j.jpsychires.2015.07.008 PMC4524806 26228427

[8] Galderisi S Rucci P Kirkpatrick B Mucci A Gibertoni D Rocca P. Interplay among psychopathologic variables, personal resources, context-related factors, and real-life functioning in individuals with schizophrenia: A network analysis. JAMA Psychiatry. (2018) 4:396–404. doi: 10.1001/jamapsychiatry.2017.4607 PMC5875306 29450447

[9] Rosen C. Periods of recovery in deficit syndrome schizophrenia: A 20-year multi–follow-up longitudinal study. Schizophr Bull. (2009) 36:788–99. doi: 10.1093/schbul/sbn167 PMC2894588 19095758

[10] Paolo FP Evangelos P Daniel S Matteo R William C Sukhwinder S. Treatments of negative symptoms in schizophrenia: meta-analysis of 168 randomized placebo-controlled trials. Schizophr Bull. (2015) 4:892. doi: 10.1093/schbul/sbu170 PMC4466178 25528757

[11] Yu M Dai ZJ Tang XW Wang X Zhang XB Sha WW. Convergence and divergence of brain network dysfunction in deficit and non-deficit schizophrenia. Schizophr Bull. (2017) 43:1315–28. doi: 10.1093/schbul/sbx014 PMC5737538 29036672

[12] Dandan Wang YW Chen Y Yu L Wu Z Liu R Ren J. Differences in inflammatory marker profiles and cognitive functioning between deficit and nondeficit schizophrenia. Front Immunol. (2022) 13:958972. doi: 10.3389/fimmu.2022.958972 36341400 PMC9627304

[13] Marder SR. The brief negative symptom scale: psychometric properties. Schizophr Bull. (2011) 37:300. doi: 10.1093/schbul/sbq059 20558531 PMC3044634

[14] Forbes C Blanchard JJ Bennett M Horan WP Kring A Gur R. Initial development and preliminary validation of a new negative symptom measure: The Clinical Assessment Interview for Negative Symptoms (CAINS). Schizophr Res. (2010) 124:36–42. doi: 10.1016/j.schres.2010.08.039 20869848 PMC2981616

[15] Blanchard JJ Cohen AS. The structure of negative symptoms within schizophrenia: implications for assessment. Schizophr Bull. (2006) 32:238–45. doi: 10.1093/schbul/sbj013 PMC2632211 16254064

[16] Cai R Huang C Ni L Liu Z Zhang S Qiu Y. The motivation and pleasure deficits but not expressivity affects social functioning through cognitive function in male patients with schizophrenia: A structural equation model. Asian J Psychiatry. (2023) 85:103616. doi: 10.1016/j.ajp.2023.103616 37163944

[17] Fang J Lv Y Xie Y Tang X Zhang X Wang X. Polygenic effects on brain functional endophenotype for deficit and non-deficit schizophrenia. Schizophrenia. (2024) 10:18. doi: 10.1038/s41537-024-00432-w 38365896 PMC10873412

[18] Strauss GP Alicia NE Ahmed AO Barchard KA Eric G Brian K. The latent structure of negative symptoms in schizophrenia. JAMA Psychiatry. (2018) 75:1271–79. doi: 10.1001/jamapsychiatry.2018.2475 PMC6583036 30208377

[19] Ahmed A Kirkpatrick B Galderisi S Mucci A Rossi A Bertolino A. Cross-cultural validation of the 5-factor structure of negative symptoms in schizophrenia. Schizophr Bull. (2019) 45:305–14. doi: 10.1093/schbul/sby050 PMC6403061 29912473

[20] Eunju J Kiho P Eunbyeol L Gregory P S Kee-Hong C. Validation of the Brief Negative Symptom Scale in Korean patients with schizophrenia. Asia-Pac Psychiatry. (2020) 12:e12382. doi: 10.1111/appy.12382 31960582

[21] Strauss GP Esfahlani FZ Galderisi S Mucci A Rossi A Bucci P. Network analysis reveals the latent structure of negative symptoms in schizophrenia. Schizophr Bull. (2019) 45:1033–41. doi: 10.1093/schbul/sby133 PMC6737465 30256991

[22] Shaffer JJ Peterson MJ McMahon MA Bizzell J Calhoun V v. Erp TGM. Neural correlates of schizophrenia negative symptoms: distinct subtypes impact dissociable brain circuits. Mol neuropsych. (2015) 1:191–200. doi: 10.1159/000440979 PMC4996000 27606313

[23] Kahn RS Sommer IE. The neurobiology and treatment of first-episode schizophrenia. Mol Psychiatry. (2014) 20:84–97. doi: 10.1038/mp.2014.66 PMC4320288 25048005

[24] Charernboon T. Interplay among positive and negative symptoms, neurocognition, social cognition, and functioning in clinically stable patients with schizophrenia: a network analysis. F1000Research. (2021) 10:1258. doi: 10.12688/f1000research.74385.3 35464178 PMC9021676

[25] Santesteban-Echarri O Paino M Rice S González-Blanch C McGorry P Gleeson J. Predictors of functional recovery in first-episode psychosis: A systematic review and meta-analysis of longitudinal studies. Clin Psychol Rev. (2017) 58:59–75. doi: 10.1016/j.cpr.2017.09.007 29042139

[26] Kirkpatrick B Fenton W Carpenter W Marder SR. The NIMH-MATRICS consensus statement on negative symptoms. Schizophr Bull. (2006) 32:214–19. doi: 10.1093/schbul/sbj053 PMC2632223 16481659

[27] Epskamp S Borsboom D Fried EI. Estimating psychological networks and their accuracy: A tutorial paper. Behav Res Methods. (2018) 50:195–212. doi: 10.3758/s13428-017-0862-1 28342071 PMC5809547

[28] Ma R Jones PJ0 McNally RJ. Bridge centrality: A network approach to understanding comorbidity. Multivar Behav Res. (2021) 56:353–67. doi: 10.1080/00273171.2019.1614898 31179765

[29] Giuliani L Sanmarchi F Mucci A Rucci P Caporusso E Bucci P. Investigating the causal pathways among psychopathological variables, cognitive impairment, and real-life functioning in people with schizophrenia. Schizophrenia. (2025) 11:1. doi: 10.1038/s41537-024-00545-2 39753575 PMC11698981

[30] Paola Rucci EC Sanmarchi F Giordano GM Mucci A Giuliani L Pezzella P. The structure stability of negative symptoms: longitudinal network analysis of the Brief Negative Symptom Scale in people with schizophrenia. BJPsych Open. (2023) 9:e168. doi: 10.1192/bjo.2023.541 37674282 PMC10594087

[31] Freeman HB Lee J. Sex differences in cognition in schizophrenia: what we know and what we do not know. Curr Top Behav Neurosci. (2023) 63:463–74. doi: 10.1007/7854_2022_394 36271194

[32] Wang X Yao SQ Fan XH Yi YQ Zhu WA Yi JY. The Chinese Version of the Schedule for the Deficit Syndrome: reliability and validity. Chin J Clin Psychol. (2005) 13:392–5. doi: 10.3969/j.issn.1005-3611.2005.04.005

[33] Galderisi S Maj M. Deficit schizophrenia: An overview of clinical, biological and treatment aspects. Eur Psychiatry. (2009) 24:493–500. doi: 10.1016/j.eurpsy.2009.03.001 19553087

[34] Kirkpatrick B Buchanan RW Mckenny PD Alphs LD Carpenter WT. The Schedule for the Deficit syndrome: an instrument for research in schizophrenia. Psychiatry Res. (1989) 30:119–23. doi: 10.1016/0165-1781(89)90153-4 2616682

[35] Sánchez-Cubillo I Periáñez J Adrover-Roig D Rodríguez-Sánchez J Ríos-Lago M Tirapu J. Construct validity of the Trail Making Test: Role of task-switching, working memory, inhibition/interference control, and visuomotor abilities. J Int Neuropsychol Soc. (2009) 15:438–50. doi: 10.1017/S1355617709090626 19402930

[36] Wolinsky F Weg MV Howren M Jones M Dotson M. A randomized controlled trial of cognitive training using a visual speed of processing intervention in middle aged and older adults. PloS One. (2013) 8:1–11. doi: 10.1371/journal.pone.0061624 PMC3641082 23650501

[37] Periáñez J Lubrini G García-Gutiérrez A Ríos-Lago M. Construct validity of the stroop color-word test: influence of speed of visual search, verbal fluency, working memory, cognitive flexibility, and conflict monitoring. Arch Clin Neuropsychol. (2020) 36:99–111. doi: 10.1093/arclin/acaa034 32514527

[38] Chaodang Z Shuchun J. A self-designed scale for social function in in-patients with psychosis (SSFPI):preliminary test of reliability and validity. Sichuan Ment Health. (2004) 3:144–6. doi: 10.3969/j.issn.1007-3256.2004.03.006

[39] Yang J Xiaokang S Lijuan W Xuezhen L Yuedi L Guozhen S. The influence of group management on social function of patients with schizophrenia. Chin Nurs Manage. (2015) 15:750–3. doi: 10.3969/j.issn.1672-1756.2015.06.034

[40] Denny Borsboom and Angélique OJ. Cramer: Network analysis: An integrative approach to the structure of psychopathology. Annu Rev Clin Psychol. (2013) 9:91–121. doi: 10.1146/annurev-clinpsy-050212-185608 23537483

[41] Dalege J Borsboom D Harreveld Fv Maas HLJvd. Network analysis on attitudes: A brief tutorial. Soc psychol Pers Sci. (2017) 8:528–37. doi: 10.1177/1948550617709827 PMC5582642 28919944

[42] Ju Gao RJ Tang X Chen J Yu M Zhou C Wang X. A neuromarker for deficit syndrome in schizophrenia from a combination of structural and functional magnetic resonance imaging. CNS Neurosci Ther. (2023) 29:1. doi: 10.1111/cns.14297 PMC10651988 37288482

[43] Fischer BA Keller WR Arango C Pearlson GD McMahon RP Meyer WA. Cortical structural abnormalities in deficit versus nondeficit schizophrenia. Schizophr Res. (2012) 136:51–4. doi: 10.1016/j.schres.2012.01.030 PMC3298625 22336954

[44] Zhou C Tang X Yu M Zhang H Zhang X Gao J. Convergent and divergent genes expression profiles associated with brain-wide functional connectome dysfunction in deficit and non-deficit schizophrenia. Trans Psychiatry. (2024) 14:124. doi: 10.1038/s41398-024-02827-w PMC10899251 38413564

[45] Der-Avakian A Markou A. The neurobiology of anhedonia and other reward-related deficits. Trends Neurosci. (2012) 35:68–77. doi: 10.1016/j.tins.2011.11.005 22177980 PMC3253139

[46] Meehl PE. Primary and secondary hypohedonia. J Psychopathol Clin Sci. (2001) 110:188–93. doi: 10.1037//0021-843x.110.1.188 11261394

[47] Blanchard JJ Mueser KT Bellack AS. Anhedonia, positive and negative affect, and social functioning in schizophrenia. Schizophr Bull. (1998) 24:413–24. doi: 10.1093/oxfordjournals.schbul.a033336 9718633

[48] Ordaz SJ Goyer MS Ho TC Singh MK Gotlib IH. Network basis of suicidal ideation in depressed adolescents. J Affect Disord. (2018) 226:92–9. doi: 10.1016/j.jad.2017.09.021 PMC12758917 28968564

[49] Swartz MS Perkins DO Stroup TS Davis SM Capuano G Rosenheck RA. Effects of antipsychotic medications on psychosocial functioning in patients with chronic schizophrenia: Findings from the NIMH CATIE Study. Am J Psychiatry. (2007) 164:428–36. doi: 10.1176/ajp.2007.164.3.428 17329467

[50] Bearden CE Rosso IM Hollister JM Sanchez LE Hadley T Cannon TD. : A prospective cohort study of childhood behavioral deviance and language abnormalities as predictors of adult schizophrenia. Schizophr Bulletin-PsycArt. (2000) 26:395–410. doi: 10.1093/oxfordjournals.schbul.a033461 10885639

[51] Häfner H Löffler W Maurer K Hambrecht M an der Heiden W. Depression, negative symptoms, social stagnation and social decline in the early course of schizophrenia. Acta Psych Scand. (1999) 100:105–18. doi: 10.1111/j.1600-0447.1999.tb10831.x 10480196

[52] Niendam TA Bearden CE Zinberg J Johnson JK O’Brien M Cannon TD. The course of neurocognition and social functioning in individuals at ultra high risk for psychosis. Schizophr Bull. (2007) 33:772–81. doi: 10.1093/schbul/sbm020 PMC2526130 17420177

[53] Costantini G Richetin J Preti E Casini E Epskamp S Perugini M. Stability and variability of personality networks. A tutorial on recent developments in network psychometrics. Pers Individ Dif. (2019) 136:68–78. doi: 10.1016/j.paid.2017.06.011

[54] Bekhuis E Schoevers R d. Boer M Peen J Dekker J Van H. Symptom-specific effects of psychotherapy versus combined therapy in the treatment of mild to moderate depression: A network approach. Psychother psychosom. (2018) 87:121–3. doi: 10.1159/000486793 PMC5969070 29495015

