# Supplementary figures and images for: Relationship between negative symptoms, cognitive function and social function in schizophrenia: new insight from a network analysis
Source: Front Psychiatry. 2025 Jun 26;16:1623147. doi: 10.3389/fpsyt.2025.1623147 (PMC12241092; doi:10.3389/fpsyt.2025.1623147)

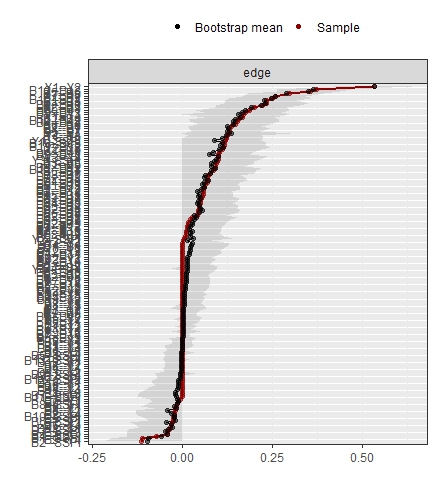

Supplement: Supplementary Figure 1 — Results of the accuracy of edge weights. [file Image1.jpeg]

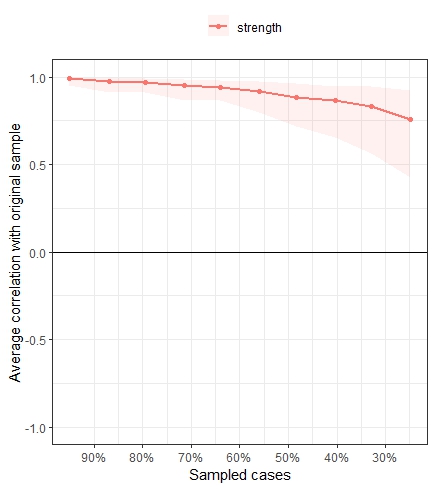

Supplement: Supplementary Figure 2 — The stability of network structure by case-dropping bootstrap. (A) stability of strength centrality; (B) stability of edge centrality; (C) stability of bridge strength. [file Image2.jpeg]

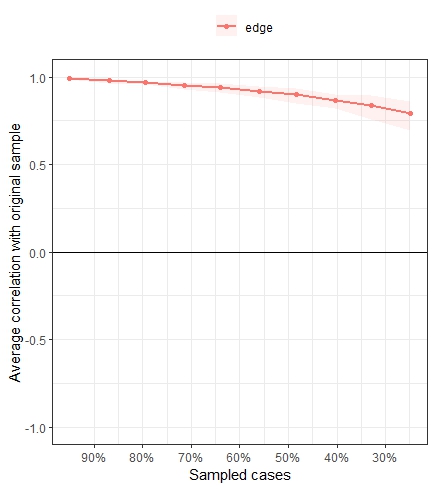

Supplement: Supplementary file 3 [file Image3.jpeg]

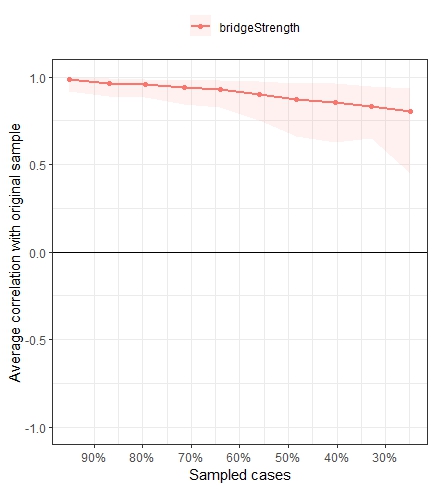

Supplement: Supplementary file 4 [file Image4.jpeg]
